# Low-intensity pulsed ultrasound enhances neurite growth in serum-starved human neuroblastoma cells

**DOI:** 10.3389/fnins.2023.1269267

**Published:** 2023-11-20

**Authors:** Xuanjie Ye, Zitong Wang, Rebekah van Bruggen, Xin-Min Li, Yanbo Zhang, Jie Chen

**Affiliations:** ^1^Department of Electrical and Computer Engineering, University of Alberta, Edmonton, AB, Canada; ^2^Department of Psychiatry, Faculty of Medicine and Dentistry, University of Alberta, Edmonton, AB, Canada

**Keywords:** low-intensity pulsed ultrasound, ultrasound parameters, neurite outgrowth, serum-starved cell model, SK-N-SH cells

## Abstract

**Introduction:**

Low-intensity pulsed ultrasound (LIPUS) is a recognized tool for promoting nerve regeneration and repair; however, the intracellular mechanisms of LIPUS stimulation remain underexplored.

**Method:**

The present study delves into the effects of varying LIPUS parameters, namely duty cycle, spatial average-temporal average (SATA) intensity, and ultrasound amplitude, on the therapeutic efficacy using SK-N-SH cells cultured in serum-starved conditions. Four distinct LIPUS settings were employed: (A) 50 mW/cm^2^, 40%, (B) 25 mW/cm^2^, 10%, (C) 50 mW/cm^2^, 20%, and (D) 25 mW/cm^2^, 10%.

**Results:**

Immunochemistry analysis exhibited neurite outgrowth promotion in all LIPUS-treated groups except for Group D. Further, LIPUS treatment was found to successfully promote brain-derived neurotrophic factor (BDNF) expression and enhance the phosphorylation of extracellular signal-regulated kinase (ERK)1/2, protein kinase B (Akt), and mammalian target of rapamycin (mTOR) signaling pathways, as evidenced by western blot analysis.

**Discussion:**

The study suggests that the parameter combination of LIPUS determines the therapeutic efficacy of LIPUS. Future investigations should aim to optimize these parameters for different cell types and settings and delve deeper into the cellular response mechanism to LIPUS treatment. Such advancements may aid in tailoring LIPUS treatment strategies to specific therapeutic needs.

## Highlights

- Our study reveals the capacity of LIPUS to promote nerve regeneration and repair.
- By systematically exploring the effects of varying LIPUS parameters on SK-NSH neuroblastoma cells, our research uncovers crucial mechanisms that underscore the therapeutic efficacy of LIPUS.
- These findings are profoundly important for neurological treatment, particularly considering the pervasive challenge of nerve regeneration in various neurodegenerative conditions.
- The capacity of LIPUS to stimulate neuroblastoma cells and facilitate neurite outgrowth could be harnessed for therapies targeting neurodegenerative diseases such as Alzheimer’s, Parkinson’s, and others.
- This approach could also be applicable in recovery after neurotrauma.

## 1 Introduction

Neurological disorders are often associated with severe consequences, impacting the affected individuals and exerting a substantial burden on the healthcare system and society (D’Andrea et al., 2003; Winkler et al., 2011; Wen and Huse, 2017; Lizarraga-Valderrama, 2021). Prevalent conditions such as traumatic brain and spinal cord injuries, cerebrovascular incidents, Alzheimer’s disease, and peripheral nerve injuries significantly reduce a patient’s quality of life (Seddon, 1942, 1943; McKhann et al., 1984; Bramlett and Dietrich, 2007; Bains and Hall, 2012; Smajlović, 2015; Scheltens et al., 2016). Numerous studies have highlighted the importance of promoting nerve regeneration and repair as a solution to recover impaired nerve functionality (Schwob, 2002; Steward et al., 2013). Consequently, considerable interest has converged toward investigating effective therapeutic strategies to enhance neural repair and regeneration.

A range of potential interventions is emerging with advances in neurobiology and related technologies. These encompass but are not limited to, stem cell therapy (Lavorato et al., 2021), gene therapy (Müller et al., 2006), utilization of biomaterials (Subramanian et al., 2009; Joung et al., 2020), and electrical stimulation (Gordon and English, 2016; Zhang et al., 2021). Supported by experimental evidence, these innovative therapeutic approaches show promise for managing neurological disorders. A further step is understanding the molecular and cellular mechanisms associated with these therapeutic approaches that control nerve repair and regeneration, which is critical to developing treatment protocols (Scheib and Höke, 2013).

Low-intensity pulsed ultrasound (LIPUS) has recently become a safe and effective method in non-invasive physical therapy, significantly advancing in various treatment areas (Khanna et al., 2009; Zhao et al., 2012; Shaheen et al., 2013). It is postulated that the therapeutic efficacy of LIPUS is based on the mechanical and non-thermal influences of ultrasound waves, leading to biologically beneficial effects within the intra- and extracellular environment (Khanna et al., 2009). Evidence shows that LIPUS can help improve bone healing and bone density recovery in cases of fractures. Similarly, LIPUS has demonstrated commendable therapeutic outcomes on soft tissue injuries (Lai et al., 2021), wound healing (Iwanabe et al., 2016), inflammation (Nakao et al., 2014), tooth-root healing (Ang et al., 2010), and others (Zhao et al., 2014; Jiang et al., 2019; Huang et al., 2020).

In addition to the aforementioned applications, accumulated evidence has revealed the vital role of LIPUS in promoting nerve regeneration and repair. For instance, a study conducted by Zhao et al. (2016) demonstrated the efficacy of combining LIPUS and nerve growth factor (NGF) in promoting neurite outgrowth via mechanotransduction-mediated extracellular signal-regulated kinase (ERK)1/2-CREB-Trx-1 signaling pathways. In addition, Han et al. (2020) reported that LIPUS can enhance the regeneration of injured dorsal root ganglion neurons through mTOR upregulation. Interestingly, mTOR has been recognized as a vital regulator in neuronal development and plasticity by participating in multiple signaling pathways, whereby disturbed mTOR signaling correlates with abnormal neuronal function and failure of many cellular processes (Licausi et al., 2010; Archer et al., 2018). Therefore, these findings underscore the considerable promise of LIPUS as a potential therapeutic strategy for neural regeneration and repair.

Despite the numerous advancements made by LIPUS in treating neurological disorders, several key issues must be addressed before clinical translation. One of these challenges is the determination of specific LIPUS parameters, including the ultrasound fundamental frequency (UFF), pulse repetition frequency (PRF), spatial average-temporal average (SATA) intensity, and duty cycle (DC). Optimal parameter settings may differ according to specific neurological conditions. Moreover, the molecular and cellular mechanisms underlying the therapeutic effects of LIPUS in neurological disorders remain inadequately explored, and conclusive assertions have yet to be established (Miller et al., 2012). Therefore, future research endeavors should focus on identifying the optimal ultrasound parameters for various neurological disorders and exploring the therapeutic mechanisms of LIPUS.

To explore the effects of LIPUS on the nervous system, we conducted an *in vitro* study utilizing SK-N-SH cells in a serum-starved environment as the experimental model. The SK-N-SH cells are derived from a human neuroblastoma cell line. Extensively employed in neurobiological research, they serve as an archetypal model for the nervous system (Green et al., 1996; Wang et al., 2007; Zhou et al., 2021; Journal of Healthcare Engineering, 2023). Our study aimed to evaluate the influence of LIPUS on neural cell growth and its interaction with protein signaling pathways. The serum-deprivation model is frequently employed as an *in vitro* injury model due to its ability to generate oxidative stress and disrupt protein expression (White et al., 2020). It has become a standard approach in numerous prior studies focusing on therapeutic development and investigating the mechanisms of recovery (Zhao et al., 2016). Building upon existing research, we utilized the serum-starved model to examine the positive influences of LIPUS on neuronal growth and uncover the related biochemical mechanisms. Specifically, we focused on proteins related to the growth and proliferation of neurons, encompassing the mammalian target of rapamycin (mTOR), ERK1/2, Protein kinase B (also known as Akt), and brain-derived neurotrophic factor (BDNF). mTOR is a serine/threonine protein kinase, functioning as a core regulator of cell growth, metabolism, and protein synthesis, and playing an essential role in neural development, synaptic plasticity, and memory formation (Saxton and Sabatini, 2017). ERK, a subset of mitogen-activated protein kinases (MAPKs), governs a range of cellular processes such as cell survival, proliferation, and differentiation, and is integral to neuronal plasticity and long-term memory formation (Roskoski, 2012). Akt is a serine/threonine kinase implicated in regulating cell survival, growth, and metabolism, and its dysregulation is linked to various neurological disorders (Manning et al., 1995). BDNF, a neurotrophin, promotes the survival of existing neurons and stimulates the growth, differentiation, and synaptic plasticity of new neurons (Bramham and Messaoudi, 2005). In addition to investigating these signaling pathways, we also examined different ultrasound parameters to identify optimal conditions for LIPUS treatment. Overall, this research sought to explore the therapeutic mechanisms of LIPUS and its effective parameters.

## 2 Materials and methods

### 2.1 Customized miniaturized LIPUS driver system

**FIGURE 1:**
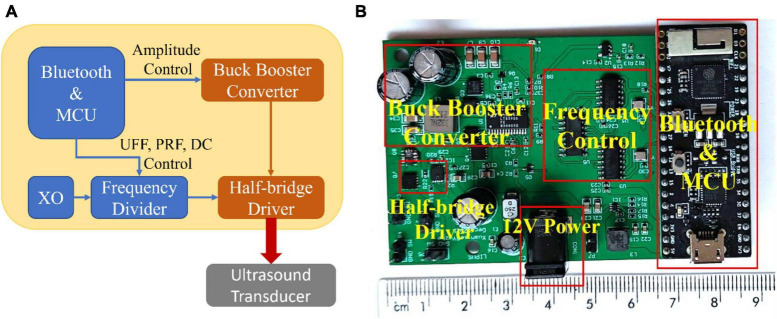
The customized miniature LIPUS driver system. **(A)** The circuit block diagram of the customized miniaturized LIPUS driver system and **(B)** the prototype.

In this study, we engineered a customized miniaturized LIPUS driver system. Figure 1A depicts the overall circuit block diagram, while Figure 1B displays the prototype. The solution for the Bluetooth communication and microcontroller unit (MCU) control module was implemented using the ESP32-PICO-KIT V4 development board [Espressif Systems (Shanghai) Co., Ltd., China]. The MCU output signal controls the output amplitude of the buck-boost converter (5-40V), UFF, PRF, and DC. The buck-boost converter is based on the synchronous 4-switch buck-boost DC/DC controller IC chip LT8390A. The system employs a half-bridge driver to drive the transducers, composed of a half-bridge gate driver IC chip DGD05463FN-7 and a dual N-channel MOSFET IC chip NTTFD4D0N04HLTWG. This device facilitates precise adjustment of ultrasound amplitude, UFF, PRF, and DC, thus allowing for quick and simple configuration and delivery of a wide array of LIPUS parameters.

### 2.2 LIPUS exposure system

The well-on-transducer method was employed for the *in vitro* ultrasound therapy, where each well was coupled with a planar transducer via a gel medium. This setup is prevalent in cell and tissue sample ultrasound studies due to its simplicity (Hensel et al., 2011). The coupling layer, composed of gel or water, facilitates acoustic matching, ensuring optimal energy transfer from the transducer to the sample. Customized ultrasound transducers for this experiment have a single resonant frequency of 1.5 MHz and a 25 mm diameter. Before each use, the SATA intensity of the ultrasound transducer was quantified utilizing the ultrasound power meter UPM-DT-1000PA (OHMIC Instruments, MO, USA). The ultrasound coupling agent and the base of the cell culture well were attached to the ultrasound transducer so that the penetrating loss of ultrasound energy would be included in the measurement.

**FIGURE 2:**
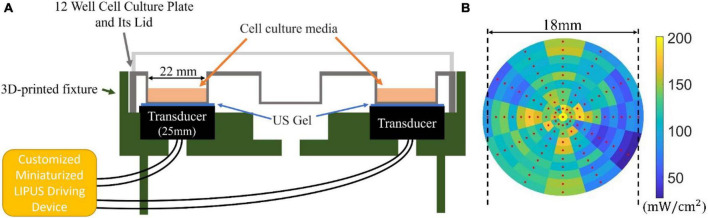
**(A)** Sketch of the LIPUS exposure setup. A 12-well culture plate (well diameter of 22 mm) was placed on a 3D-printed base. Four 25 mm transducers placed in the four corners of the plate contacted the well through ultrasound (US) gel medium. The transducers were connected to the customized miniaturized LIPUS driving device. **(B)** The ultrasonic sound field distribution measured from the bottom of the well (SATA intensity 125 mW/cm^2^ with a duty cycle of 100%).

A 12-well cell culture plate, arranged in a 3 × 4 matrix, was used for the cell culture. Each well had a diameter of 22 mm, smaller than the ultrasound transducer, which exposed all cells within the well to the LIPUS. The cells were seeded in the wells located at the four corners. This enabled simultaneous LIPUS treatment across four wells, enhancing experimental efficiency. The unoccupied central wells minimized ultrasound crosstalk between neighboring wells. To assure efficient ultrasound transmission, an ultrasound gel (Wavelength^®^ MP Blue Multi-Purpose Ultrasound Gel, ON, Canada) was used as the coupling medium between the ultrasound transducer and the cell culture plate, as depicted in Figure 2A.

To measure the ultrasonic field intensity distribution at the well bottom, a customized 3D-printed jig was developed for accurate hydrophone (HNR-1000, Onda Corporation, CA, USA) placement. The sampling procedure commenced from the center of the well, systematically moving outward at 30° intervals and sampling every 1 mm in the radial direction. Each sampled point represented the local pressure distribution, which was subsequently converted into power intensity using the equation $I=\frac{p_{0}^{2}}{2\,z}$, where *I*, *p_0_*, *z* stands for the power intensity, ultrasonic pressure, and acoustic impedance, respectively. During testing, the ultrasound system was adjusted to output a constant sound field SATA intensity of 125 mW/cm^2^ at a duty cycle of 100%, as measured by an ultrasound power meter UPM-DT-1000PA. This resulted in a SATA intensity of 25 mW/cm^2^ by adjusting the duty cycle to 20%. The outcome of our measurements is presented in Figure 2B, where the red dot signifies the location of the test point. Although some variations in the sound field distribution were observed, leading to higher (200 mW/cm^2^) and lower (50 mW/cm^2^) intensity areas, the majority of the tested regions remained from 100 to 150 mW/cm^2^.

### 2.3 Cell culture and LIPUS treatment

**FIGURE 3:**
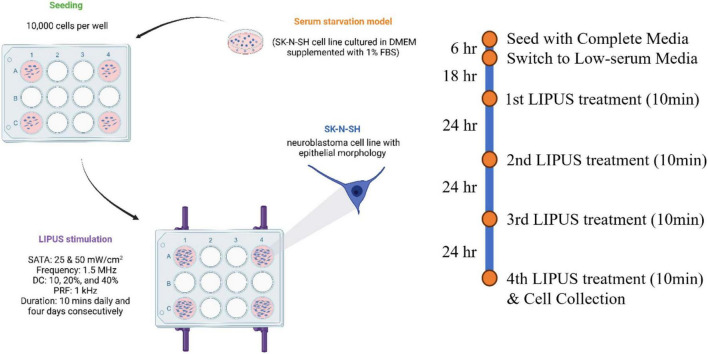
LIPUS stimulation protocol. Serum-starved SK-N-SH cells were treated with 10 min of LIPUS stimulation daily for four consecutive days (UFF: 1.5 MHz; PRF: 1 kHz; SATA intensity: either 25 or 50 mW/cm^2^; DC: either 10, 20, or 40%).

The overall LIPUS stimulation protocol is shown in Figure 3. The SK-N-SH cell line was kindly provided by Dr. Tom Hobman, Department of Cell Biology, Faculty of Medicine and Dentistry, University of Alberta, Edmonton, Canada. SK-N-SH cells were cultured in Dulbecco’s Modified Eagle Medium (DMEM; 319-005-CL, WISENT INC.) with 10% fetal bovine serum (FBS; 090150, WISENT INC.) within a humidified 37°C incubator. This culture medium is referred to as complete media. For seeding, SK-N-SH cell suspension was adjusted to a concentration of 10,000 cells/mL, and each well was loaded with 1 mL of the suspension, resulting in an initial seeding density of 10,000 cells per well. Approximately 6 h later, following adherence of the majority of cells to the well surface, the culture medium was aspirated and the wells were washed 3 times with DMEM without FBS. The culture medium was then replaced with a low-serum media, composed of DMEM supplemented with 1% FBS, creating a serum-starved cell model.

Two control groups were included: (1) SK-N-SH cells cultured in complete media (10% FBS) without LIPUS stimulation were used as a healthy control group, and (2) SK-N-SH cells grown in low-serum media without LIPUS stimulation were used as the control for serum starvation. Four LIPUS treatment groups grown in low-serum media (1% FBS) were tested. To investigate the effect of various SATA intensities (mW/cm^2^) and DC (%) on cellular responses, four distinct LIPUS treatment parameter configurations were tested: (A) 50 mW/cm^2^, 40%, (B) 25 mW/cm^2^, 20%, (C) 50 mW/cm^2^, 20%, and (D) 25 mW/cm^2^, 10%. Groups A and C shared the same ultrasound intensity but varied in duty cycles. This pattern was mirrored in groups B and D. Notably, the ultrasound amplitude in groups B and D was approximately 0.707 times that of groups A and C. UFF was set at 1.5 MHz and the PRF was set to 1 kHz in the four groups and remained constant. LIPUS treatment began 18 h post-transition to the low-serum media (on day 2) for 10 min. To ensure a uniform ultrasound distribution, the transducer was rotated 180° following the initial 5-min treatment period, then continued for 5 min. The 10-min LIPUS treatment was repeated every 24 h. Ten minutes after the fourth LIPUS treatment (on day 5), the supernatants were collected for cell viability assessments, and the cells were either collected for protein extraction or imaged using immunocytochemistry (ICC).

### 2.4 Cell cytotoxicity quantification

The LDH-Cytotoxicity Assay Kit II (ab65393) was utilized to quantify lactate dehydrogenase (LDH) release to indicate cytotoxicity. Cell supernatants were collected and cleared of debris via centrifugation (600×g). Following the manufacturer’s instructions, a water-soluble tetrazolium (WST) substrate mix was added to the cleared supernatant and mixed thoroughly in a 96-well plate. Following a 30-min incubation at room temperature, the absorbance was measured at 450 nm, with a reference wavelength of 650 nm using a colorimetric microplate reader (FLUOstar Omega Microplate Reader, BMG). A low LDH control, composed of cell-free media, and a high LDH control, composed of the supernatant of the SK-N-SH cells cultured in the complete media for 5 days, were used to calculate cell cytotoxicity. Cytotoxicity (%) was calculated using the following calculation: Cytotoxicity = (Test Sample – Low Control)/(High Control – Low Control) × 100%. Results were normalized to the cytotoxicity of the healthy control group.

### 2.5 Cell visualization

ICC was employed to visualize cellular structures to observe cell morphology. For ICC experiments, glass coverslips were washed, sterilized, and placed in the 12-well cell culture plate before SK-N-SH seeding. Cells were treated as outlined above. On the fifth day of the experiment (after the fourth LIPUS treatment), cells were rinsed once with phosphate-buffered saline (PBS; 311-010-CL, WISENT INC.) and then fixed in a solution of 4% paraformaldehyde (PFA; 441244, Sigma-Aldrich) diluted in 1 × PBS. This fixation process was carried out at room temperature for 10 min. Subsequently, cells were rinsed twice with 1 × PBS. Cell membranes were permeabilized using 0.2% Triton X-100 (A16046, Thermo Fisher Scientific) diluted in 1 × PBS, for 5 min at room temperature, followed by three washes with 1 × PBS. The coverslips were then transferred onto a strip of parafilm in a humidifying chamber and incubated with blocking buffer (0.5% bovine serum albumin (BSA; A2134, Biomatik) and 6% normal goat serum (ab7481, Abcam) diluted in 1× PBS) for 1 h at room temperature. Following this, primary antibodies (Tubulin, 1:500, MAB1637, Sigma-Aldrich) diluted in a 1:1 mixture of blocking buffer and 1 × PBS were added to the samples and incubated either for 2 h at room temperature or overnight at 4°C. The coverslips were then washed three times for 5 min in 1 × PBS and incubated with the secondary antibody solution [goat anti-mouse (1:1000, Alexa Fluor 594, A-11032, Invitrogen)] diluted in 1 × PBS for 1 h at room temperature. Subsequently, the secondary antibody solution was aspirated, and the coverslips were rinsed three times with 1 × PBS. The coverslips were then mounted using ProLong Gold antifade reagent with DAPI (P36935; Invitrogen) and were allowed to dry overnight at room temperature, protected from light. Finally, the ICC imaging was conducted using the EVOS M5000 Imaging system (Thermo Fisher Scientific Inc., MA, USA).

### 2.6 Western blot

Western blot analysis was utilized to quantify BDNF, ERK1/2, Akt, and mTOR expression levels. Cells for Western blot analysis were removed from the plate wells using Trypsin-EDTA (0.05% with phenol red, 325-043-EL, Wisent) and resuspended in DMEM with 10% FBS. Cells were collected via centrifugation (1000 rpm, 10 min) and then lysed using RIPA buffer (150 mM NaCl, 25 mM Tris-HCl pH 7.6, 5 mM EDTA, 1% Triton X-100, 0.1% SDS, 1% sodium deoxycholate) mixed with phosphate inhibitor (A32957, Thermo Scientific) and protease inhibitor (A32963, Thermo Scientific). Protein lysates were quantified using the Bradford protein assay kit (Bio-Rad). The resultant absorbance was measured with the FLUOstar Omega Microplate Reader (BMG). Subsequently, 5 μg of each protein sample was loaded onto a hand-cast 10% Sodium Dodecyl Sulfate Polyacrylamide Gel Electrophoresis (SDS-PAGE) gel. Proteins were transferred onto a 0.2 μm nitrocellulose membrane (1620112, Bio-Rad). The membranes were blocked using 5% BSA in 1 × Tris-Buffered Saline (TBS) and incubated in the desired primary antibody solution overnight at 4°C. Then, membranes were washed and probed with secondary antibodies at room temperature for 1 h. Protein bands were visualized using the imaging system, with the images captured and quantified using Image Studio Lite software (LI-COR Biosciences, Lincoln, NE, Ver 5.2). Glyceraldehyde 3-phosphate dehydrogenase (GAPDH) was utilized as a loading control to ensure equal sample loading. The band intensities were quantified using ImageJ. The relative band intensities were normalized as follows: BDNF normalized to GAPDH, phosphorylated Akt (p-Akt) to total Akt, phosphorylated ERK1/2 (p-ERK1/2) to total ERK1/2, and phosphorylated mTOR (p-mTOR) to total mTOR.

The primary antibodies used in the study were diluted 1:1000 with 5% BSA in 1 × TBS: rabbit anti-BDNF (ab108319, Abcam), rabbit anti-ERK1/2 (9102S, Cell Signaling), rabbit anti-p-ERK1/2 (4376S, Cell Signaling), mouse anti-Akt (1:1000; 2920S, Cell Signaling), rabbit anti-p-Akt (4060S, Cell Signaling), rabbit anti-mTOR (2983S, Cell Signaling), rabbit anti-p-mTOR (5536S, Cell Signaling), and mouse anti-GAPDH (1:1000; 97166S, Cell Signaling). The secondary antibodies were diluted 1:10000 with 5% BSA in 1 × TBS: goat anti-rabbit-IR800 (926-32211, LI-COR Biosciences) and goat anti-mouse-IR680 (926-68070, LI-COR Biosciences).

### 2.7 Statistical analysis

Statistical analysis was conducted on data for LDH quantification results, neurite length, soma size, and western blot quantification outcomes using GraphPad Prism software (GraphPad Software, MA, USA, version 9.4). The comparison among the healthy control, serum-starved control, and LIPUS-treatment groups was based on the one-way analysis of variance (ANOVA) followed by a *post hoc* multiple comparison test. The observed power for each analysis was calculated to ascertain that the sample size was sufficient to substantiate the findings. Statistical significance was designated at a *p*-value threshold of less than 0.05.

## 3 Results

### 3.1 Cytotoxicity results

**FIGURE 4:**
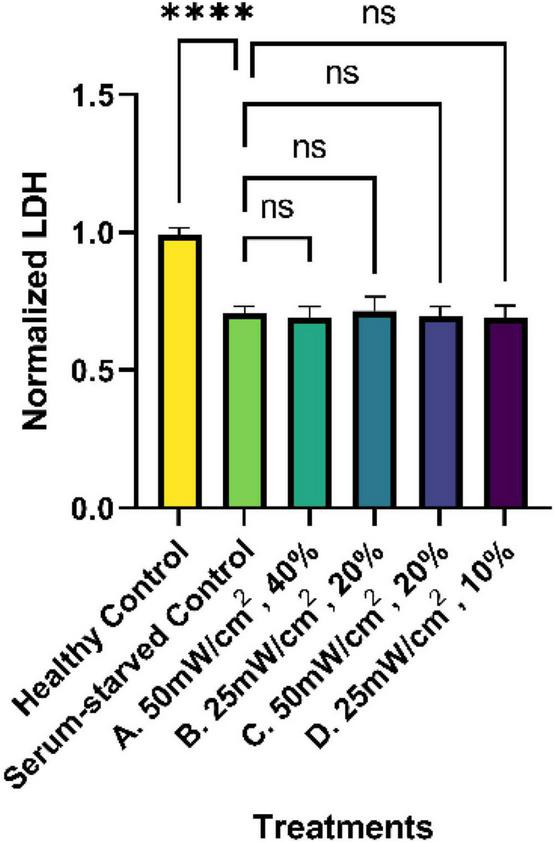
Normalized percent cytotoxicity from SK-N-SH cells treated with LIPUS. LDH levels from the supernatant of untreated (healthy control and serum-starved control) and LIPUS-treated SK-N-SH cells were quantified using a commercially available LDH assay. All results were normalized to the healthy control group. *N* = 5/group. ns: no statistical significance; ****p < 0.0001.

To determine if the LIPUS treatment altered cell viability, LDH levels from all treatment groups were evaluated as a measure of cytotoxicity. Dying cells release LDH, which can be measured using a commercially available kit. Analysis of the cytotoxicity assay revealed that LDH levels in all serum-starved groups (serum-starved control and LIPUS treatment groups A, B, C, D), were significantly lower than the healthy (complete media) control with a value of roughly 0.7 (*p* < 0.0001), as presented in Figure 4. However, no statistical significance was observed among the serum-starved control group and the LIPUS-treatment groups A, B, C, and D, suggesting that the LIPUS treatments did not alter cell survival in serum-deprived conditions.

### 3.2 ICC results

**FIGURE 5:**
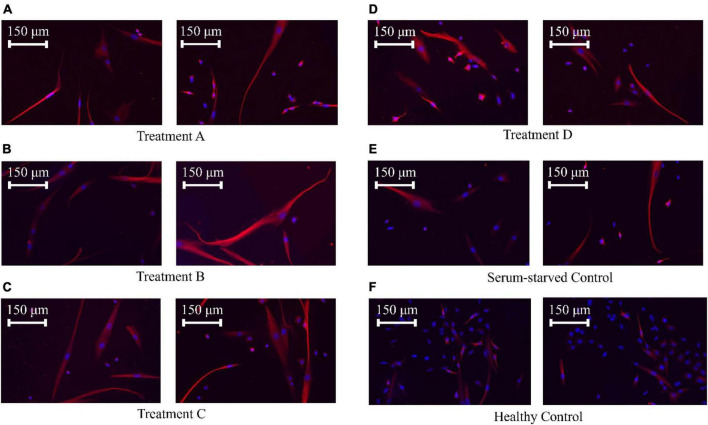
SK-N-SK cell ICC staining. Nuclei were stained with DAPI (blue), and microtubules, which represent the cytoskeleton, were stained with an anti-tubulin antibody (red). **(A)** SK-N-SH cells treated with LIPUS (50 mW/cm^2^, 40%). **(B)** SK-N-SH cells treated with LIPUS (25 mW/cm^2^, 20%). **(C)** SK-N-SH cells treated with LIPUS (50 mW/cm^2^, 20%). **(D)** SK-N-SH cells treated with LIPUS (25 mW/cm^2^, 10%). **(E)** Serum-starved control. **(F)** Healthy control.

An in-depth analysis of neurite lengths in the serum-starved control and LIPUS-treatment groups was performed to understand how different LIPUS settings can influence nerve cell growth. Representative ICC images of each group are shown in Figure 5. The lengths of the neurites were measured using NeuronJ, an ImageJ plugin tailored for neurite tracing and analysis. In the analysis, we exclusively focused on the longest neurite of each cell, typically considered the axon. The length of the axon is crucial for nerve signal transmission and the overall function of the nerve cell (Debanne et al., 2011). Additionally, we measured the width of the cell body at its widest point. Any neurite lengths less than the diameter of the cell body were excluded as they were considered inadequately developed neurites. As the healthy control group exhibited a high cell density with most of the neurites densely interwoven, neurite length was not quantified. To facilitate statistical analysis, neurite length was quantified from the edge of the cell nucleus (DAPI) to the distal end of the cell skeleton (Tubulin) using the NeuronJ. The measurements were conducted following the guidelines provided by NeuronJ, and default parameters were employed for the neurite length measurements.

**FIGURE 6:**
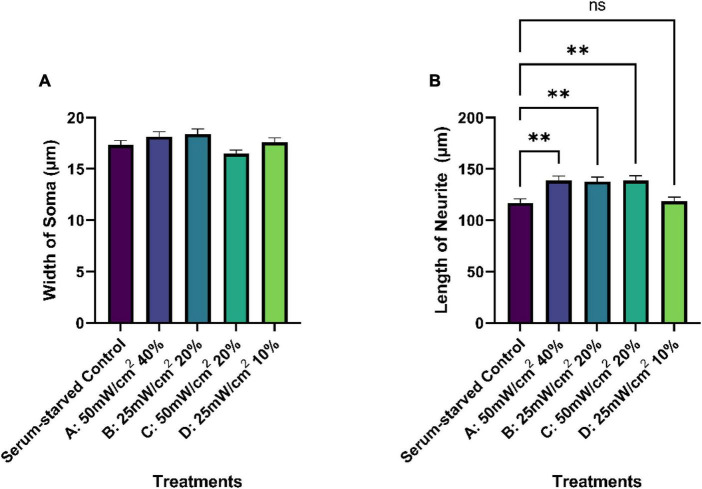
Quantification of average soma width **(A)** and neurite length (μm) **(B)** of serum-starved SK-N-SH cells under different treatment conditions (control and LIPUS treatment, Groups A-D). *N* = 250. Ns, no statistica significance; **p < 0.005.

No significant differences were observed among the cell body/soma diameters between SK-N-SH cells in control or LIPUS treatment groups under low-serum conditions, as presented in Figure 6A. However, LIPUS-treatment groups A, B, and C, exhibited a significant enhancement in neurite growth compared to the serum-starved control group (*p* < 0.005), whereby the mean ± standard error length for each group was as follows: serum-starved control group 117.1 ± 57.0 μm, Group A 138.9 ± 63.5 μm, Group B 137.7 ± 64.1 μm, and Group C 138.7 ± 70.5 μm, as shown in Figure 6B. The LIPUS-treatment group D (25 mW/cm^2^, 10%) did not show a significant difference in neurite length (mean ± standard error neurite length: 118.6 ± 60.8 μm) compared to the serum-starved control group.

### 3.3 Western blot results

**FIGURE 7:**
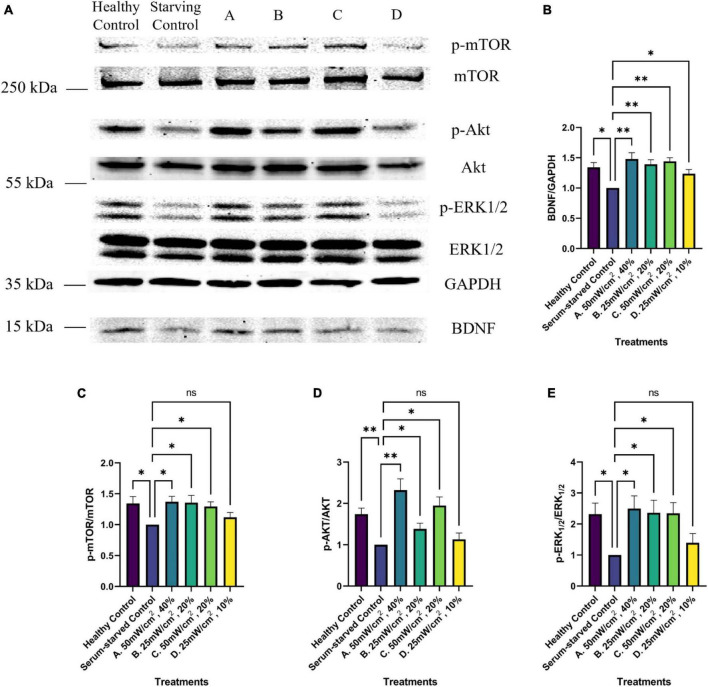
Western blot analysis of BDNF growth factor signaling pathway activation upon LIPUS stimulation. **(A)** Representative Western Blot imaging results of GAPDH, BDNF, p-mTOR, mTOR, p-ERK1/2, ERK1/2, p-Akt, and Akt from untreated (healthy control, Serum-starved Control) and LIPUS-treated (A, B, C, D) SK-N-SH cells. *N* = 5 replicates per group. Comparative analysis of normalized Western blot protein band intensity of panel **(B)** BDNF, **(C)** mTOR, **(D)** Akt, and **(E)** ERK1/2 across various treatment groups (*N* = 5). *p < 0.05, **p < 0.01, ns, no statistical significance.

Western blot analyses were conducted to evaluate BDNF levels and the activation of the BDNF signaling pathway, namely by assessing the phosphorylation status of ERK, Akt, and mTOR, in response to the LIPUS treatments. Serum starvation significantly decreased BDNF levels compared to cells grown in complete media. However, this decrease was ameliorated by three of the four LIPUS treatment parameters (*p* < 0.01, Figure 7). SK-N-SH cells treated with a SATA of 50 mW/cm^2^ (Groups A and C) or with a SATA of 25 mW/cm^2^ group and the higher duty cycle of 20% (Group B) exhibited increased BDNF levels. Notably, while the Group D treatment parameters (SATA 25 mW/cm^2^, 10% DC) significantly upregulated BDNF levels, these parameters failed to elicit significant elevation in downstream signaling events. Increased BDNF levels were associated with an increase in phosphorylation levels of Akt, ERK1/2, and mTOR for LIPUS-treatment groups A, B, and C, though not group D (Figure 6), suggesting that these pathways were activated in the SK-N-SH cells upon treatment.

## 4 Discussion

Previous research has demonstrated that LIPUS can stimulate nerve regeneration and neurite outgrowth through the ERK1/2 and mTOR signaling pathways (Miller et al., 2012; Lv et al., 2015; Jiang et al., 2016; Sato et al., 2016; Zhao et al., 2016, 2017; Han et al., 2020). This study sought to determine the optimal intensity and duty cycle of ultrasound stimulation on serum-deprived SK-N-SH cells and explore the underlying molecular mechanisms under LIPUS stimulation.

In cell culture, serum is an important source of nutrients and growth hormones. Decreasing the serum level in media can induce a starvation state, which can alter cell signaling and growth. In this study, SK-N-SH cells were cultured in serum-starved conditions for 4 days, leading to limited nutrient availability. This resulted in a reduced metabolic state and slower cell division due to nutrient deprivation. Compared to cells in complete media (healthy control), those in serum-starvation conditions exhibited slower growth, as evident from the ICC analysis and cytotoxicity levels. In addition, lower cell growth rates were observed alongside reduced BDNF levels and decreased activation of the Akt, ERK1/2, and mTOR pathways compared to healthy controls. These findings confirm the successful establishment of the experimental starvation model. Interestingly, LIPUS stimulation under the tested conditions had no significant impact on cell proliferation or cytotoxicity when compared to the serum-starved control group. Furthermore, LIPUS treatment did not affect the soma size of SK-N-SH cells. Altogether, these results suggest that the LIPUS treatment does not impact neuronal proliferation – either negatively or positively.

**FIGURE 8:**
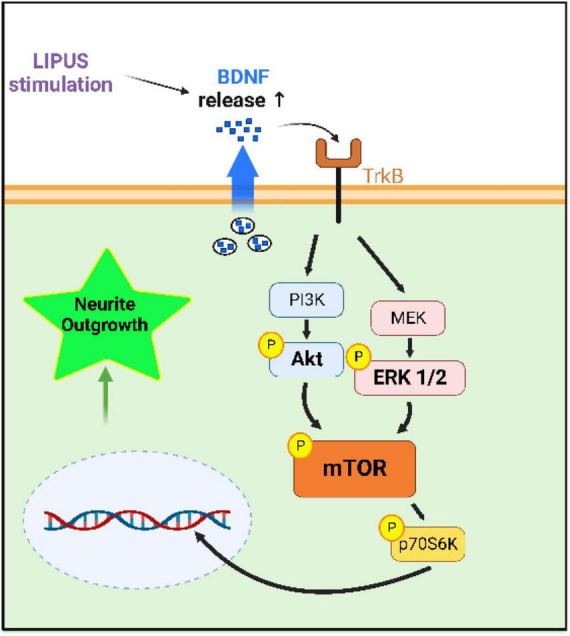
Hypothesized mechanism by which LIPUS stimulates neurite outgrowth. LIPUS stimulation increases the release of the growth factor BDNF, activating the mTOR pathway downstream of Akt and ERK1/2, leading to neurite outgrowth.

While the LIPUS treatment appeared to have no impact on cell proliferation, enhanced neurite growth was found in three out of the four tested LIPUS treatments. In addition, the three LIPUS conditions (A, B, C) leading to increased neurite length were associated with higher BDNF expression and increased phosphorylation of ERK1/2, Akt, and mTOR, compared to the serum-starved control. Previous studies have highlighted the pivotal roles of the ERK1/2 and Akt signaling pathways in mediating the effects of growth factors such as nerve growth factor and BDNF on neuronal growth under LIPUS treatment (Zhao et al., 2016; Guo et al., 2021). Additionally, evidence supports the role of LIPUS in promoting the regeneration of injured dorsal root ganglion neurons through activation of the mTOR pathway (Han et al., 2020). This study replicates these findings, demonstrating the upregulation of these proliferation-related proteins in nerve cells stimulated by LIPUS, and proposes a comprehensive mechanistic pathway, as depicted in Figure 8. The discrepancy in p-Akt levels among conditions A, B, and C can be attributed to variable responses induced by different LIPUS parameters. While condition B showed lower p-Akt levels compared to A and C, it still elevated p-Akt levels compared to the serum-starved control. Moreover, the consistent expression of p-mTOR across conditions suggests that ERK1/2 may play a predominant role in mTOR regulation within LIPUS therapy.

This study investigated the effects of four distinct ultrasound parameter settings on SK-N-SH cells cultured in a low-serum environment. It is widely believed that the therapeutic effects of LIPUS arise mainly from non-thermal effects, including cavitation and mechanical effects (Snehota et al., 2020). Our experimental results corroborated this perspective, underscoring that the therapeutic efficacy of LIPUS is not primarily attributable to its thermal effects. Within the LIPUS treatment groups, groups B and D shared identical SATA intensities, indicating a similar thermal influence. Nonetheless, while group B exhibited enhanced neurite growth associated with increased BDNF signaling, group D displayed no significant deviation from the serum-starved control group. Notably, although group B had only half the thermal effect of groups A and C, they showed a similar promoting effect on neurite outgrowth. Overall, these findings suggest that the non-thermal effects of LIPUS, rather than the thermal effects, play a more significant role in promoting neurite growth and altering protein signaling pathway expression. Moreover, from a safety perspective in the context of LIPUS treatment, group B, possessing only half the SATA power of groups A and C, consistently demonstrated a comparable enhancement in neurite growth. This observation underscores the potential of parameter B (25 mW/cm^2^, 20%) as a safer choice for both future research endeavors and clinical applications.

Future studies are required to systematically investigate the effects of different LIPUS parameters, encompassing duty cycle, SATA intensity, and ultrasound amplitude, on therapeutic efficacy. The comparison between group D and other groups reveals that slight adjustments in these parameters can significantly alter the therapeutic effect of LIPUS. Understanding the contribution of these parameters to the overall treatment effect is crucial for developing more effective LIPUS treatment strategies. In addition, in-depth studies of the molecular and cellular processes that control the observed therapeutic effects are also needed. Expanding knowledge of the underlying mechanisms and interactions among LIPUS parameters would enable researchers and clinicians to tailor LIPUS therapies to specific therapeutic needs.

## 5 Conclusion

This study investigated the effects of various LIPUS parameters on SK-N-SH cells cultured in serum-starved conditions. Four parameter settings were studied, altering either the SATA (mW/cm^2^) or the duty cycle (%): A (50 mW/cm^2^, 40%), B (25 mW/cm^2^, 10%), C (50 mW/cm^2^, 20%), and D (25 mW/cm^2^, 10%). ICC results revealed that parameter groups A, B, and C stimulated neurite outgrowth, associated with increased BDNF expression and enhanced the phosphorylation of ERK1/2, Akt, and mTOR signaling pathways. The investigation also revealed that the combination of SATA intensity, duty cycle, and ultrasound amplitude critically determined the therapeutic efficacy of LIPUS, which appears unrelated to any thermal effects of ultrasound. Future research is required to optimize different parameters for various cell types and experimental settings, and explore the in-depth mechanism of cellular response to LIPUS treatment. These advancements will help researchers and clinicians tailor LIPUS treatment strategies to specific treatment needs.

## Data availability statement

The original contributions presented in this study are included in this article/supplementary material, further inquiries can be directed to the corresponding authors.

## Ethics statement

Ethical approval was not required for the studies on humans in accordance with the local legislation and institutional requirements because only commercially available established cell lines were used.

## Author contributions

XY: Conceptualization, Data curation, Formal analysis, Investigation, Methodology, Software, Validation, Visualization, Writing – original draft, Writing – review and editing. ZW: Conceptualization, Investigation, Methodology, Software, Validation, Visualization, Writing – review and editing. RB: Formal analysis, Investigation, Methodology, Writing – review and editing. X-ML: Funding acquisition, Resources, Supervision, Writing – review and editing. YZ: Funding acquisition, Project administration, Supervision, Writing – review and editing. JC: Conceptualization, Funding acquisition, Project administration, Resources, Supervision, Writing – review and editing.
